# Advances in Quantitative Techniques for Mapping RNA Modifications

**DOI:** 10.3390/life15121888

**Published:** 2025-12-10

**Authors:** Ling Tian, Bharathi Vallabhaneni, Yie-Hwa Chang

**Affiliations:** 1 Mediomics, LLC, 5445 Highland Park Drive, Saint Louis, MO 63110, USA; tianl@mediomics.com (L.T.); vallabhanenib@mediomics.com (B.V.); 2 Edward A. Doisy Department of Biochemistry and Molecular Biology, Saint Louis University School of Medicine, Saint Louis, MO 63104, USA

**Keywords:** RNA modifications, epitranscriptomics, mass spectrometry, chemical-assisted sequencing, nanopore direct RNA sequencing, single-cell analysis, single-molecule imaging, biomarker discovery

## Abstract

RNA modifications are essential regulators of gene expression and cellular function, modulating RNA stability, splicing, translation, and localization. Dysregulation of these modifications has been linked to cancer, neurodegenerative disorders, viral infections, and other diseases. Precise quantification and mapping of RNA modifications are crucial for understanding their biological roles. This review summarizes current and emerging methodologies for RNA modification analysis, including mass spectrometry, antibody-based and non-antibody-based approaches, PCR- and NMR-based detection, chemical- and enzyme-assisted sequencing, and nanopore direct RNA sequencing. We also highlight advanced techniques for single-cell and single-molecule imaging, enabling the study of modification dynamics and cellular heterogeneity. The advantages, limitations, and challenges of each method are discussed, providing a framework for selecting appropriate analytical strategies. Future perspectives emphasize high-throughput, multiplexed, and single-cell approaches, integrating multiple technologies to decode the epitranscriptome. These approaches form a robust toolkit for uncovering RNA modification functions, discovering biomarkers, and developing novel therapeutic strategies.

## 1. Introduction

RNA modifications—co-transcriptional or post-transcriptional chemical alterations of RNA molecules—play pivotal roles in regulating gene expression and maintaining cellular homeostasis. Since the discovery of the first RNA modification, pseudouridine (Ψ), in 1957 [1], more than 170 distinct modifications have been identified across various RNA species in all three domains of life [2]. These chemical marks are widespread, occurring not only in abundant non-coding RNAs such as ribosomal RNA (rRNA), transfer RNA (tRNA), and small nuclear RNA (snRNA), but also in messenger RNA (mRNA), highlighting their broad and versatile regulatory functions.

RNA modifications exhibit remarkable chemical diversity, ranging from simple methylations to more complex modifications such as acetylation and thiolation. They are dynamically regulated by “writer,” “eraser,” and “reader” proteins, which, respectively, catalyze, remove, and interpret these chemical marks. Among the most extensively studied modifications are N^6^-methyladenosine (m^6^A), inosine (I), pseudouridine (Ψ), 5-methyluridine (m^5^U), 5-methylcytosine (m^5^C), and N^1^-methyladenosine (m^1^A) [3,4]. These modifications influence fundamental aspects of RNA metabolism, including stability, splicing, translation efficiency, and subcellular localization. Importantly, aberrant regulation of RNA modifications has been associated with a wide range of diseases, including cancer, diabetes, cardiovascular and neurodegenerative disorders, and viral infections. The growing recognition of their biological significance has driven intense efforts to develop accurate and sensitive methods for their detection, quantification, and mapping.

**Figure 1:**
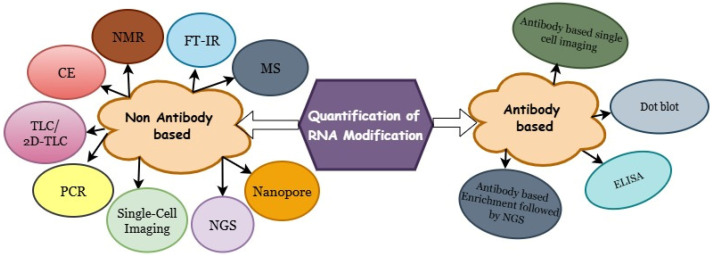
Overview of traditional and emerging techniques for quantifying RNA modifications.

Quantification and mapping of RNA modifications are essential for elucidating their biological functions, as these analyses reveal their abundance, spatial distribution, and potential regulatory impact on gene expression. Classical biochemical methods, such as dot blot assays, thin-layer chromatography (TLC), and mass spectrometry (MS), have long served as reliable tools for detecting and quantifying RNA modifications, providing valuable information about their global abundance and chemical composition. However, advances in high-throughput sequencing technologies have transformed the field, enabling transcriptome-wide and site-specific detection of RNA modifications. Methods such as RNA bisulfite sequencing, m^6^A-seq, and nanopore direct RNA sequencing now allow quantification of modifications at single-nucleotide resolution. More recently, technological innovations have made it possible to map specific RNA modifications at single-cell resolution, offering new insights into cell-type-specific epitranscriptomics landscapes. In this review, we provide a comprehensive overview of both traditional and emerging strategies for the quantification of RNA modifications (Figure 1), encompassing mass spectrometry–based, sequencing-based, and imaging-based methods. We also highlight recent advances not covered in previous reviews [5,6,7,8] and discuss their implications for the expanding field of epitranscriptomics.

## 2. Methods for RNA Quantification and Imaging

### 2.1. Antibody-Based Methods

**Figure 2:**
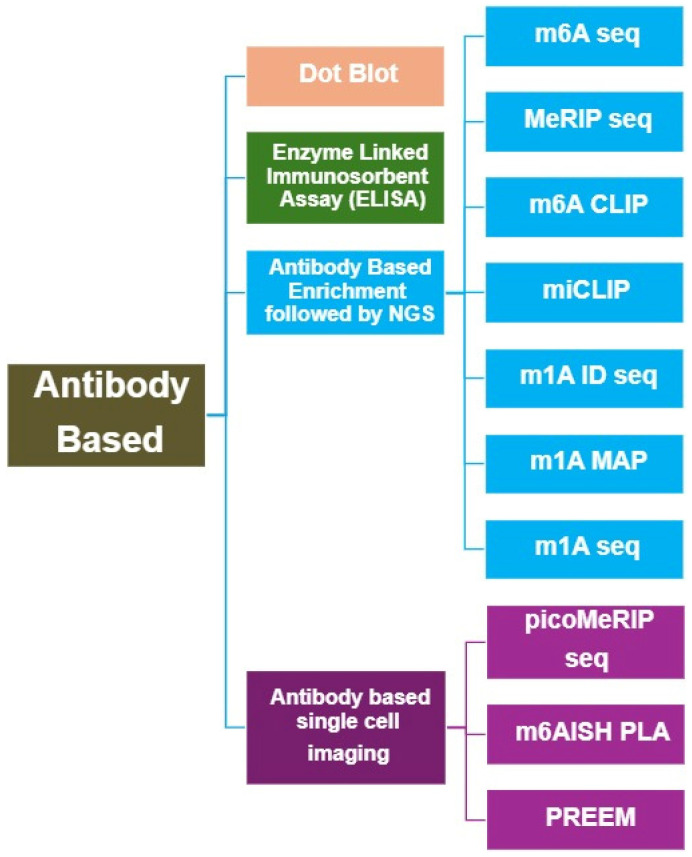
Overview of Antibody-based methods for RNA Modification Analysis.

Specific antibodies for detecting RNA modifications were first proposed and successfully implemented in the late 1970s [9]. Since then, the development of high-affinity, modification-specific antibodies has greatly advanced the ability to detect, enrich, and map RNA modifications across different RNA species (Table 1). Antibody-based methods remain among the most widely used experimental strategies for RNA modification analysis due to their simplicity, versatility, and compatibility with various downstream applications (Figure 2).

#### 2.1.1. Dot Blot

The dot blot is a simple and widely used method for detecting and semi-quantifying RNA modifications. In this technique, RNA samples are directly spotted onto a membrane—typically nitrocellulose or polyvinylidene fluoride (PVDF)—without electrophoretic separation. The membrane is then probed with a modification-specific primary antibody, followed by a labeled secondary antibody for signal detection. This cost-effective assay has been employed to detect several RNA modifications, including Ψ [10], m^5^C [10,11], hm^5^C [12,13], and m^6^A [14,15], in both coding and non-coding RNA species. Mishima et al. introduced the “immuno-northern blot”, a modified Northern blotting technique that employs antibodies specific to modified nucleosides [16]. This approach allows RNAs to be separated based on their molecular weights and is used to detect modifications such as m^1^A, m^6^A, Ψ, and m^5^C in total RNA from mammalian cells, yeast, and bacteria. While the method provides semi-quantitative data and is effective for detecting highly abundant modifications, its sensitivity is constrained by antibody specificity and the amount of input RNA. Moreover, these methods offer only semi-quantitative data and lack single-nucleotide or locus-level resolution, restricting their applicability for detailed transcriptome-wide analyses.

#### 2.1.2. Enzyme-Linked Immunosorbent Assay (ELISA)

The enzyme-linked immunosorbent assay (ELISA) is another widely adopted antibody-based technique for quantifying RNA modifications. In this assay, modified RNA or synthetic standards are immobilized on microplates and compete with endogenous RNA for the binding to modification-specific antibodies. Detection is typically achieved using a horseradish peroxidase (HRP)-conjugated or fluorophore-labeled secondary antibody, generating colorimetric or fluorescent signals proportional to the level of bound antibody. D’Ambrosio et al. [17] developed a highly sensitive ELISA for m^1^I detection in human urine, achieving nanogram-level sensitivity. More recently, Ensinck et al. [18] introduced a rapid m^6^A-ELISA protocol capable of detecting dynamic m^6^A changes in yeast and mouse embryonic stem cells (ESCs) using as little as 25 ng of mRNA, with the entire assay completed in under one day. Commercial colorimetric ELISA kits are now available for multiple RNA modifications, including m^6^A, m^1^A, m^5^C, and I.

Although ELISA provides high sensitivity and convenience, its quantification accuracy may decline at very low analyte concentrations. Therefore, complementary high-resolution techniques such as liquid chromatography–tandem mass spectrometry (LC–MS/MS), polymerase chain reaction (PCR)-based assays, or sequencing-based methods are often required for ultra-sensitive or site-specific detection.

#### 2.1.3. Antibody-Based Enrichment Coupled with Next-Generation Sequencing (NGS)

Immunoprecipitation followed by high-throughput sequencing has revolutionized RNA modification mapping. In these methods, modification-specific antibodies selectively enrich RNA fragments containing the targeted modification prior to sequencing. This strategy was first implemented for m^6^A with the introduction of m^6^A-seq [19] and methylated RNA immunoprecipitation sequencing (MeRIP-seq) [20]. Both approaches fragment RNA into ~100–200 nucleotide segments, use anti-m^6^A antibodies for enrichment, and sequence the immunoprecipitated fragments. These pioneering studies identified over 12,000 m^6^A peaks in human and mouse mRNA. Similarly, MeRIP-Seq mapped 7676 m^6^A-modified genes in mammals.

However, early antibody-based sequencing lacked single-nucleotide resolution due to limited antibody specificity and imprecise RNA fragmentation. To improve resolution, UV crosslinking was incorporated to create covalent bonds between antibodies and modified RNA sites. Techniques such as m^6^A-CLIP [21] and methylation individual-nucleotide-resolution crosslinking and immunoprecipitation (miCLIP) [22] exploit reverse transcription (RT) truncations or misincorporations (e.g., C-to-T transitions) to pinpoint modification sites at single-nucleotide resolution. miCLIP also enables discrimination between m^6^A and its derivative m^6^Am by analyzing distinct RT signatures [23]. Similar immunoprecipitation-based sequencing approaches have been developed for other modifications, including m^1^A (m^1^A-ID-seq and m^1^A-MAP) [24,25,26], hm^5^C [13], ac^4^C [27], and m^7^G [28]. Nevertheless, antibody-dependent methods often suffer from cross-reactivity, low affinity, and biases introduced during UV crosslinking or RT. For example, anti-m^1^A antibodies can cross-react with m^6^A, resulting in false positives [29]. Moreover, the requirement for RNA fragmentation limits detection to modification-dense regions. Emerging chemical-assisted and direct RNA sequencing methods—such as DART-seq and nanopore-based detection—aim to overcome these limitations and achieve unbiased, base-resolution mapping. DART-seq avoids antibody enrichment, UV crosslinking, and RNA fragmentation by enzymatically marking m^6^A sites, thereby reducing crosslinking-associated RNA damage and RT-derived artifacts.

#### 2.1.4. Antibody-Based Single-Cell Imaging of RNA Modifications

To extend antibody-based methods to low-input and single-cell applications, Li et al. [30] developed picoMeRIP-seq, a picogram-scale m^6^A immunoprecipitation and sequencing approach. Unlike conventional MeRIP-seq, picoMeRIP-seq optimizes RNA fragmentation, recovery, and library preparation to enable m^6^A mapping from minimal input samples, including individual zebrafish zygotes, mouse oocytes, and preimplantation embryos. This method enables in vivo analysis of m^6^A distribution without specialized instrumentation, making it suitable for rare or clinically relevant samples.

For spatial visualization, Li’s group further developed m^6^A in situ hybridization–mediated proximity ligation assay (m^6^AISH-PLA) [31]. This method combines fluorescence in situ hybridization (FISH) probes with m^6^A-specific antibodies, enabling single-cell and single-molecule visualization of m^6^A modifications through rolling circle amplification. Applied to HSP70 mRNA under heat shock, m^6^AISH-PLA revealed dynamic cytoplasmic redistribution of transcripts. Although the method is low-throughput and cannot always resolve closely spaced m^6^A sites, it represents an important advance in RNA modification imaging. Recently, Zhu and colleagues introduced PREEM (PRoximity Exchange-assisted Encoding of Multichrome), a high-resolution imaging method for m^6^A detection at single-cell and single-molecule resolution [32]. PREEM employs Boolean “AND” logic recognition combined with hybridization chain reaction (HCR) amplification to visualize multiple m^6^A sites simultaneously using limited imaging channels. This innovative technique enhances quantitative and spatial mapping of m^6^A, revealing cell-to-cell variability and dynamic epitranscriptomic responses to external stimuli.

### 2.2. Non-Antibody-Based Methods

**Figure 3:**
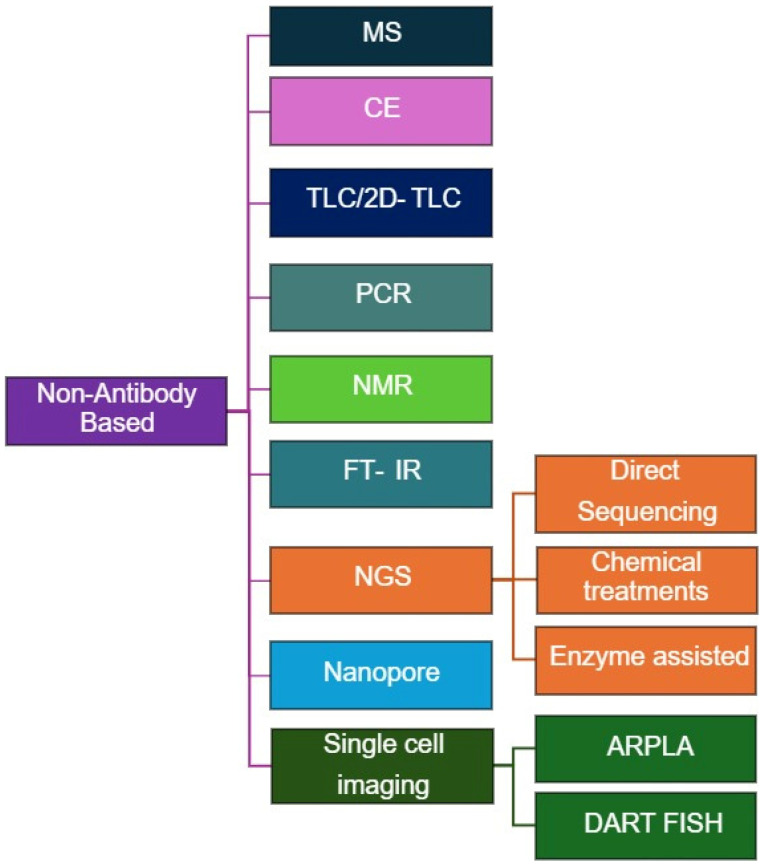
Overview of Non-Antibody-based methods for RNA Modification Analysis.

Non-antibody-based methods for RNA modification quantification (Figure 3) provide direct, high-resolution, and sometimes label-free alternatives to antibody-based assays. These techniques exploit chemical, enzymatic, or physical differences between modified and unmodified nucleotides, enabling quantitative and site-specific detection across diverse RNA species (Table 2). Although certain antibody-based approaches may incorporate mass-spectrometry (MS) for detection or validation, in this review we classify MS-based techniques under non-antibody–based methods for clarity and organizational consistency.

#### 2.2.1. Mass Spectrometry (MS)-Based Methods

Mass spectrometry (MS) has emerged as a cornerstone technology in epitranscriptomics, enabling the identification, quantification, and mapping of RNA modifications with exceptional accuracy. MS directly detects mass changes resulting from chemical modifications, providing a precise and unbiased readout of nucleoside alterations. Low-resolution instruments, such as quadrupole analyzers, offer high sensitivity for detecting trace-level modifications, while high-resolution platforms—including time-of-flight (TOF) and Orbitrap systems—provide accurate mass measurements, elemental composition analysis, and structural elucidation through fragmentation. Pioneered over two decades ago by McCloskey and colleagues [33], MS-based approaches have become indispensable for studying RNA modifications. Two main MS strategies—bottom-up and top-down mapping—are commonly applied for the identification and localization of modifications within known RNA sequences [34].

##### Bottom-Up MS Approach

The bottom-up approach involves enzymatic digestion of RNA into smaller oligonucleotides, followed by liquid chromatography–mass spectrometry (LC–MS) or tandem mass spectrometry (LC–MS/MS) analysis to map post-transcriptional modifications. Site-specific ribonucleases (e.g., RNase T1, RNase A, RNase U2) generate oligonucleotide fragments compatible with MS, allowing sensitive and precise identification of RNA modifications, even in complex biological samples.

In LC–MS workflows, nucleosides are separated by high-performance liquid chromatography (HPLC), ionized—typically via electrospray ionization (ESI)—and analyzed according to their mass-to-charge ratio (*m*/*z*). Distinct mass spectra and retention times enable the identification and quantification of individual nucleosides. LC–MS/MS enhances this process by adding a fragmentation step, wherein precursor ions are selected and fragmented into product ions for analysis in a second mass analyzer. This tandem approach yields structural information critical for distinguishing isomers and confirming modification identities, achieving excellent sensitivity and specificity. When combined with isotope-labeled internal standards, LC–MS/MS can reach quantification limits in the low attomole range [35,36].

Bottom-up MS is particularly suited for profiling low-abundance RNA modifications in mRNA, rRNA, and other non-coding RNAs. For example, LC–MS has been applied to map RNA modifications in rat peripheral blood and tissues, revealing tissue-specific modification patterns across mammalian epitranscriptomes [37,38]. LC–MS/MS provides a sensitive quantitative platform for detecting diverse modifications, including m^6^A [39,40], m^5^C [41], m^1^A [42], hm^5^C [43], m^1^G [44], Ψ [45,46], and novel species such as 3,2′-O-dimethylcytidine (m^3^Cm) [47], Im [48], ac^4^C [49], m^4^Cm, and 1,N^6^-dimethyladenosine (m^1^,^6^A). These discoveries have significantly expanded the catalog of known eukaryotic RNA modifications.

The clinical relevance of RNA modification profiling is increasingly evident. Cancer-associated alterations in RNA modification patterns are being explored as diagnostic and prognostic biomarkers. LC–MS/MS methods are now being validated for quantifying multiple modified nucleosides in tissue and liquid biopsy samples, revealing correlations between modification abundance and metastatic progression [50]. For example, LC–MS/MS analyses have detected elevated levels of m^7^G, 2′-O-methylcytidine (Cm), and 2′-O-methylguanosine, alongside reduced m^22^G and N^2^,N^2^,7-trimethylguanosine (m^2^,^2^,^7^G) in miRNA fractions from Alzheimer’s disease (AD) cortices, suggesting epitranscriptomic dysregulation as a hallmark of AD [51]. Similarly, LC–MS/MS has quantified m^6^A levels in severe acute respiratory syndrome coronavirus 2 (SARS-CoV-2) RNA [52], and subsequent work demonstrated that m^5^C occurs at higher abundance than m^6^A, suggesting it as a potential antiviral drug target [53].

Despite its strengths, the bottom-up approach faces several challenges. Enzymatic digestion disrupts RNA secondary structure, leading to loss of sequence context. Incomplete digestion may cause uneven fragment coverage, and co-elution of modified and canonical nucleosides during chromatography can complicate quantification. Adjusting LC conditions or employing multidimensional separation (e.g., ion mobility) can improve resolution but often requires method revalidation. Ionization bias is another limitation—ESI tends to favor polar molecules, underrepresenting hydrophobic modifications. Derivatization techniques (e.g., permethylation) or alternative ionization methods can mitigate this issue but add procedural complexity. Moreover, positional isomers (e.g., m^1^A vs. m^6^A) and mass-silent modifications (e.g., Ψ) share identical *m*/*z* values, complicating localization. While chromatographic retention time (RT) can aid resolution, it may lack reproducibility across instruments. To overcome these limitations, researchers have employed ion mobility–based MS [54] and higher-energy collisional dissociation (HCD) [55] to discriminate isomers based on their shape or fragmentation profiles.

##### Top-Down MS Approach

In contrast, the top-down MS approach analyzes intact RNA molecules and their modifications without prior enzymatic digestion. The method involves direct introduction of undigested RNA into the mass spectrometer, where gas-phase dissociation techniques fragment the RNA to generate sequence and modification information. The concept originated in the early 1990s, when MS was first used to determine molecular masses of tRNA isoacceptors and 5S rRNA [56].

Advances in instrumentation (e.g., quadrupole time-of-flight (QqTOF) and Fourier transform ion cyclotron resonance (FT-ICR) mass spectrometers) and dissociation methods such as collision-induced dissociation (CID) and electron detachment dissociation (EDD)—have extended analysis to longer RNA molecules (up to 61 nt) [57] and even full-length tRNAs [58,59]. More recently, novel fragmentation techniques such as Radical Transfer Dissociation (RTD) [60] and Activated-Ion Negative Electron Transfer Dissociation (AI-NETD) [61] have improved sequence coverage and reduced spectral complexity.

Unlike bottom-up MS, top-down MS preserves the full-length RNA structure, enabling de novo sequencing and direct mapping of mass-altering modifications without introducing enzymatic biases. It also allows the simultaneous analysis of sequence, modifications, and higher-order structural features, including RNA–protein complexes. Advanced dissociation methods such as RTD and AI-NETD reduce internal fragments and improve spectral clarity, facilitating the detection of labile modifications (e.g., 5-formylcytidine).

Nevertheless, top-down MS presents technical challenges. It requires highly purified RNA and specialized high-resolution instruments (e.g., FT-ICR or QqTOF MS). Spectrum interpretation remains complex due to the large number of fragment ions, and certain RNA regions (e.g., anticodon loops in tRNAs) may resist fragmentation, reducing sequence coverage. Despite these challenges, continuous innovations in sample preparation, derivatization, and computational data analysis are steadily enhancing the power and applicability of top-down MS in RNA modification research.

#### 2.2.2. Capillary Electrophoresis (CE)

Capillary electrophoresis (CE) is an analytical technique employing narrow-bore capillaries (20–200 μm i.d.) and high voltages to achieve efficient separation of biomolecules. When coupled with ultraviolet (UV) spectroscopy or laser-induced fluorescence (LIF), CE offers high sensitivity and rapid analysis of RNA modifications. This method enables the detection of modified nucleotides such as I, xanthosine (X), Ψ, m^2^G, m^1^A, and m^2,2^G at low micromolar concentrations in complex biological samples, including urine and tissue extracts [62,63]. Advanced CE–mass spectrometry (CE–MS) approaches further allow for detailed profiling of minor RNA modifications, including m^6^A, Am, and m^5^C, without requiring derivatization [64]. Despite its robustness, CE faces challenges in reproducibility and long-term stability, which may limit its use in high-throughput applications.

#### 2.2.3. Thin-Layer Chromatography (TLC) and Two-Dimensional TLC (2D-TLC)

Thin-layer chromatography (TLC) and two-dimensional TLC (2D-TLC) are classical analytical methods that separate nucleotides based on their differential mobilities in orthogonal solvent systems. 2D-TLC enhances resolution and has been instrumental in studying post-transcriptional RNA modifications. The technique typically involves enzymatic digestion of RNA, 5′-end radiolabeling with [γ-^32^P] ATP using T4 polynucleotide kinase, and subsequent separation on TLC plates. Modified nucleotides are identified by comparing retardation factor (Rf) values with standards and quantified by measuring radioactivity. 2D-TLC can be applied to identify various modified nucleotides in different types of RNA, including tRNA, rRNA, and mRNA [65,66,67]. Although TLC is cost-effective and widely accessible, it relies on radioactive reagents and provides semi-quantitative results. Later adaptations, such as coupling RNase H cleavage with TLC, improved quantification accuracy, as demonstrated in Ψ quantification in U2 RNA, revealing 90% pseudo-uridylation at position U34 in mouse liver RNA [68]. The SCARLET method (site-specific cleavage and radioactive labeling followed by ligation-assisted extraction and TLC) further enabled locus-specific m^6^A determination in mRNAs and lncRNAs without requiring RNA purification [69]. However, these methods remain low throughput, require prior sequence knowledge, and depend on isotopic reagents.

#### 2.2.4. Polymerase Chain Reaction (PCR)-Based Methods

Polymerase chain reaction (PCR)-based approaches, particularly quantitative PCR (qPCR), provide sensitive and specific detection of RNA modifications by exploiting their effects on reverse transcription or ligation reactions. RNA modifications such as m^6^A and m^1^A hinder Watson–Crick base pairing or stall reverse transcriptases, leading to truncated cDNA or mutation signatures that can be quantified by PCR amplification.

Liu et al. developed a ligation-based PCR strategy for m^6^A detection [70]. This approach utilizes DNA ligases and polymerases that are sensitive to RNA modifications: T3 DNA ligase fails to join probes annealed to m^6^A-containing RNA, resulting in reduced ligation efficiency. The ligation products are subsequently amplified by qPCR, with fluorescence signals reflecting m^6^A levels. Similarly, the SELECT method quantifies m^6^A by leveraging its inhibitory effects on Bst DNA polymerase elongation and SplintR ligase activity, allowing linear quantification of m^6^A fractions [71]. For m^1^A detection, Ding et al. applied a similar ligation-assisted qPCR approach. Here, m^1^A disrupts base pairing with uridine, decreasing ligation efficiency. The resulting truncated products are quantified via qPCR, enabling site-specific m^1^A detection [72]. The Reverse Transcription at Low dNTP (RTL-P) method is a sensitive approach for detecting 2′-O-methylation (Nm) sites. Nm modifications block reverse transcriptase at low dNTP concentrations but permit readthrough at high dNTP levels [73]. Nm status is inferred by comparing PCR product intensities under low- versus high-dNTP conditions. Specificity can be further enhanced using engineered DNA polymerases, such as thermostable KlenTaq variants, which selectively discriminate against Nm modifications during RT-qPCR [74].

Ψ can be detected by inducing mutations during reverse transcription. Ψ reacts with N-cyclohexyl-N′-(2-morpholinoethyl) carbodiimide (CMC), forming adducts that disrupt base pairing and cause misincorporations or deletions in cDNA. These changes alter qPCR melting curves, allowing locus-specific Ψ detection [75]. PCR-based methods offer high sensitivity, speed, and accessibility compared to labor-intensive techniques like radiolabeling or mass spectrometry. However, they require prior sequence knowledge for primer and probe design, limiting their application in de novo modification discovery.

#### 2.2.5. Nuclear Magnetic Resonance (NMR) Spectroscopy

Nuclear Magnetic Resonance (NMR) spectroscopy has been instrumental in elucidating RNA modifications, providing detailed insights into RNA structure, dynamics, and stability. Modified nucleotides generate distinct chemical shifts, making them readily identifiable in NMR spectra. Early NMR studies primarily focused on tRNA structure and folding, mapping the unique chemical shifts in modified nucleotides [76,77]. More recently, Barraud et al. [78] developed an innovative NMR approach that allows real-time tracking of tRNA modification events in cell extracts. Advances in NMR methodologies have expanded their applications beyond tRNA, enabling the identification of modifications across diverse RNA species while providing structural and dynamic insights [79].

NMR has been employed to characterize a wide range of RNA modifications, including 5-methylaminomethyluridine (mnm^5^U) [80], mnm^5^s^2^U, t^6^A, m^7^G [81], N1-methyl-N3-(3-amino-3-carboxypropyl) pseudouridine (m^1^acp^3^Ψ) [82], and m^5^U [83]. These studies have revealed critical details about RNA dynamics. For example, NMR and FRET analyses of a 32-nucleotide hairpin from the metastasis-associated lung adenocarcinoma transcript 1 (MALAT1) m^6^A -switch demonstrated that m^6^A selectively destabilizes the U5-tract, increasing solvent accessibility while maintaining the overall hairpin structure. This modification induces a conformation resembling the RNA–HNRNPC (heterogeneous nuclear ribonucleoprotein C) complex, suggesting a role in facilitating protein binding [84]. Similarly, chemical exchange saturation transfer (CEST) studies showed that m^6^A slows RNA annealing by promoting alternative base-pairing conformations [85], while m^6^A in the lncRNA Xist locally unfolds RNA stems to enhance reader protein binding [86]. Additional NMR analyses have revealed broader effects of modifications on RNA structure and dynamics: m^6^A alters base pairing and flexibility [87]; Ψ stabilizes RNA structures [88]; dihydrouridine (D) increases RNA flexibility [89]; and m1A and m1G modulate RNA conformation and stability [84]. Despite inherent limitations, such as sensitivity constraints and spectral complexity, NMR has evolved from a tool for static structural determination into a dynamic probe for studying RNA modification processes. With continued methodological advancements, NMR is poised to remain an indispensable technique in RNA epitranscriptomics research.

#### 2.2.6. FT-IR Spectroscopy

Complementary to NMR and sequencing-based strategies, Fourier-transform infrared (FT-IR) spectroscopy provides a rapid, label-free, non-antibody method to assess global RNA modification patterns [90]. FT-IR detects characteristic vibrational absorbance signatures of RNA chemical bonds, allowing observation of spectral shifts associated with prevalent modifications such as m^6^A, m^5^C, and pseudouridine. Although it does not offer site-specific resolution, FT-IR delivers a fast, minimally invasive, bulk-level readout of overall RNA modification status, making it attractive for screening or profiling total RNA methylation in low-input or partially degraded samples.

**Table 2:** Non-Antibody-Based Methods for RNA Modification Quantification.

Method	Subtypes/Mechanism	Examples of RNA Modifications Studied	References
MS	Direct chemical analysis of nucleosides after RNA digestion; detection and quantification of modified nucleotides based on mass-to-charge ratios (e.g., LC–MS, LC–MS/MS).	m^6^A, m^5^C, hm^5^C, ac^4^C, Ψ, m^1^A, m^7^G, other nucleoside-level modifications.	[33,34,35,36,37,38,39,40,41,42,43,44,45,46,47,48,49,50,51,52,53,54,55,56,57,58,59,60,61]
CE	Separates RNA fragments based on size and charge.	I, X, Ψ, m^2^G, m^1^A, m2,2G, m^6^A, Am, m^5^C	[62,63,64]
TLC/2D-TLC	Resolves RNA modifications using distinct nucleotide mobilities in orthogonal solvent systems.	Ψ, m^6^A	[65,66,67,68,69]
PCR	RNA modifications impede reverse transcription or DNA polymerase activity.	Nm, Ψ	[70,71,72,73,74,75]
NMR	Detects unique chemical shifts and coupling patterns from modified nucleotides.	mnm5U, t6A, mnm5s2U, m7G, m^1^A, m^6^A, m^1^G, m^1^acp3-Ψ, m^5^U, Ψ, D	[76,77,78,79,80,81,82,83,84,85,86,87,88,89]
NGS	Direct sequencing: RT misincorporations (I → G). Chemical treatments: Various chemical reactions generate RT signatures (e.g., NaBH_4_, NaCNBH_3_, Bromoacrylamide, CMC, aC, allyl-SeAM). Enzyme-assisted: Modification-sensitive enzymes or engineered RT introduce mutations or cleave modified nucleotides.	I, Nm, m^6^A, m^7^G, ac^4^C, Ψ, m^3^C, m^5^C	[91,92,93,94,95,96,97,98,99,100,101,102,103,104,105,106,107,108,109,110,111,112,113,114,115,116,117,118,119,120,121,122,123,124,125,126,127,128,129]
Nanopore	Direct RNA sequencing; detects modifications by analyzing disruptions in ionic current.	m^6^A, m^7^G, m^5^C, Ψ	[130,131,132,133,134,135,136,137]
Single-cell imaging	ARPLA: Sialic acid-specific aptamer + PLA-RCA. DART-FISH: Combines DART-seq with FISH for m^6^A detection.	glycoRNA, m^6^A	[138,139]

Note: Some antibody-based workflows incorporate mass spectrometry; however, for simplicity, all MS-based techniques are classified here as non–antibody-based methods.

#### 2.2.7. Next-Generation Sequencing (NGS)-Based Approaches

Next-generation sequencing (NGS) has transformed the study of RNA modifications, enabling high-throughput, transcriptome-wide analysis with unprecedented depth and accuracy. NGS-based methods typically involve converting RNA into complementary DNA (cDNA) and sequencing it to detect the presence and abundance of specific modifications. RNA modifications can alter nucleotide properties—such as base-pairing, susceptibility to enzymatic activity, or binding to specific proteins or antibodies—producing characteristic signatures during reverse transcription (RT) or sequencing [91,92]. The main strategies used in NGS-based RNA modification analysis include:

##### Direct Sequencing

Some RNA modifications naturally interfere with cDNA synthesis, causing RT truncations or misincorporations detectable in NGS data. For example, I is read as G, generating A-to-G mismatches, enabling the identification of A-to-I editing sites in transcriptomes [93,94,95]. Nm modifications stall RT enzymes under low-dNTP conditions, forming the basis of 2OMe-seq and MeTH-seq [96,97].

##### Chemical Treatment Approaches

Chemical treatments are widely used to detect RNA modifications by selectively altering modified nucleotides. These changes generate signatures during reverse transcription (RT), which can be read by next-generation sequencing (NGS) [98]. Examples include m^7^G detection: Sodium borohydride (NaBH_4_) reduces m^7^G to abasic sites, causing RT misincorporations or deletions. Methods include m7G-MaP-seq [99], BoRed-seq [100], and m7G-quant-seq [101], applied to rRNA, tRNA, and miRNA. ac^4^C detection: Sodium cyanoborohydride (NaCNBH_3_) reduces ac^4^C to N4-acetyltetrahydrocytidine under acidic conditions. ac4C-seq uses this to map temperature-dependent ac^4^C in archaea [102] and other organisms [103]. Ψ detection: Bromoacrylamide induces Ψ-to-C transitions (BACS) [104]. N-cyclohexyl-N′-(2-morpholinoethyl) carbodiimide metho-p-toluenesulfonate (CMC) forms bulky RT-blocking adducts used in Pseudo-seq [105] and CeU-seq [106]. m^6^A detection: GLORI uses glyoxal (protects G) and nitrite (converts unmethylated A to inosine) to preserve m^6^A, enabling quantitative single-nucleotide mapping across RNA types [107,108]. Inosine detection: Acrylonitrile converts inosine into ce1I, blocking RT, as used in ICE-seq [109] for A-to-I editing mapping. Ψ detection: Ψ can be chemically modified with CMC, creating a bulky adduct that stops reverse transcription. This causes truncations, which are detected in methods like Pseudo-seq and CeU-seq to map Ψ at single-nucleotide resolution [105,106].

Some approaches identify RNA modifications by exploiting chemical reactions that cleave the RNA backbone at unmodified sites and protect modified sites from cleavage, followed by selective adapter ligation and sequencing. Examples include Nm detection: RiboMeth-seq uses alkaline hydrolysis to fragment unmodified RNA while Nm-protected sites remain intact [110]. Optimized protocols allow detection with as little as ~1 ng RNA [111]. Nm-Seq and RibOxi-seq: Use periodate (NaIO_4_) oxidation to degrade unmodified 2′-OH, improving throughput and compatibility with clinical samples [112,113]. m7G and m3C detection: AlkAniline-seq uses NaBH_4_ and aniline-induced cleavage for single-nucleotide mapping [114]. Ψ detection: HydraPsi-seq exploits Ψ resistance to hydrazine/aniline cleavage to map Ψ across RNA types [115].

Other chemical-assisted sequencing approaches include, m5C: RNA-BisSeq converts unmethylated cytosine to uracil, while methylated cytosines remain unchanged [116,117]. m^6^A: NOSeq and m^6^A-ORL-seq use selective deamination/oxidation (e.g., NO, NaNO_2_) for single-nucleotide mapping [118,119]. m^6^A-label-seq incorporates N6-allyladenosine at m^6^A sites in living cells; iodine-induced cyclization generates RT signatures detectable by sequencing [120].

Chemical treatments provide a versatile toolkit to detect RNA modifications at single-nucleotide resolution. While highly precise, many methods require substantial RNA input or harsh conditions, which can limit throughput or applicability to low-abundance RNAs.

##### Enzyme-Assisted Methods

Enzyme-assisted approaches leverage modification-sensitive enzymes that selectively recognize, cleave, or induce mutations at modified nucleotides, enabling precise RNA modification mapping. For instance, Nm-Mut-seq uses an engineered reverse transcriptase to introduce mutations specifically at Am-, Cm-, and Gm-modified sites under restrictive RT conditions, allowing detection of Nm modifications even in low-abundance RNAs [121]. Endonuclease-based methods have been widely applied to map A-to-I editing. Glyoxal selectively protects guanosine but not inosine from RNase T1 digestion, enabling high-throughput identification of hundreds of A-to-I sites in mouse brain RNA [122]. Human endonuclease V (hEndoV) has been utilized in the hEndoV-seq for the single-base resolution detection of A-to-I editing sites [123]. However, Ψ and U confound the result by cross-reacting with the derivative compounds. Knutson et al. found that while Mg^2+^ enables eEndoV to catalyze RNA cleavage, Ca^2+^ instead facilitates inosine binding without cleavage, allowing high-affinity capture of Inosine in RNA. Leveraging this property, they developed EndoVIPER-seq (Endonuclease V inosine precipitation enrichment sequencing), an efficient method to enrich A-to-I edited transcripts before RNA-seq, significantly improving both the depth of sequencing coverage and the accuracy of editing site detection, outperforming traditional approaches [124]. Wei et al. developed a specific ligation of inosine-cleaved sequencing (Slic-seq) method for transcriptome-wide identification of Inosine based on EndoV cleavage activity and high reactivity of sodium periodate to RNA 3′ terminal [125]. Applying Slic-seq to human HEK293T cells, the researchers identified known and novel editing sites, demonstrating the method’s sensitivity and reliability. Notably, Slic-seq effectively detected editing sites in low-expression transcripts and regions with low editing levels, addressing the limitations of previous methodologies. RNA deaminases have also been employed for RNA modification mapping. Meyer developed DART-seq (Deamination Adjacent to RNA Modification Targets) to profile m^6^A transcriptome-wide. This method fuses the cytidine deaminase APOBEC1 to the m^6^A-binding YTH domain, inducing C-to-U deamination adjacent to m^6^A residues, which can be detected as C-to-T mutations by high-throughput sequencing [126]. Notably, DART-seq is compatible with low-input total RNA (~10 ng). This approach was extended to single-cell resolution as scDART-seq [127]. To achieve site-specific quantification, eTAM-seq and AD-seq utilize engineered TadA variants to selectively convert adenosine to inosine at m^6^A sites, distinguishing methylated from unmodified adenosines. These methods enable quantitative m^6^A mapping even from as few as ten cells [128,129]. Overall, enzyme-assisted strategies provide sensitive, specific, and scalable mapping of RNA modifications, particularly for low-abundance or single-cell samples, complementing chemical-based methods. While enzyme-assisted strategies provide sensitive, low-input, and near–single-nucleotide mapping of RNA modifications, they face challenges including sequence-context bias, off-target editing, dependency on adjacent editable bases, and quantification variability (Table 3).

#### 2.2.8. Nanopore Sequencing

Nanopore sequencing, developed by Oxford Nanopore Technologies (ONT), enables direct, single-molecule RNA sequencing without amplification or cDNA conversion, preserving RNA modifications and allowing simultaneous transcriptome-wide detection [130]. Single-stranded RNA molecules pass through a biological or synthetic nanopore under applied voltage, producing characteristic disruptions in ionic current. These signals are interpreted by machine learning-based base callers to determine both sequence and modifications [131].

This approach has successfully detected multiple RNA modifications. m^6^A is identified through systematic base-calling errors and current deviations [132,133], m7G in rRNA and tRNA is detected using modification-trained datasets [134], Ψ is inferred from dwell-time changes and signal fluctuations [135], and m5C is distinguished from unmodified cytosines via signal intensity analysis [136]. Nanopore sequencing offers long-read capability for full-length transcripts, direct detection of modifications, RNA secondary structure analysis, and real-time sequencing. Despite these advantages, challenges such as relatively high error rates [137], significant RNA input requirements, cost, and limited throughput still constrain widespread application.

#### 2.2.9. Non-Antibody-Based Single-Cell Imaging

Advanced imaging techniques such as ARPLA (Sialic Acid Aptamer and RNA In Situ Hybridization-Mediated Proximity Ligation Assay) and DART-FISH (Deamination Adjacent to RNA Modification Targets–Fluorescence In Situ Hybridization) enable single-cell visualization of RNA modifications. ARPLA uses sialic acid-specific aptamers combined with proximity ligation and rolling circle amplification to detect glycosylated RNAs on cell surfaces [138]. In contrast, DART-FISH integrates DART-seq with FISH to visualize m^6^A-modified transcripts at single-molecule resolution, revealing isoform-specific and stress-dependent modification patterns [139]. Despite throughput and accessibility limitations, these approaches represent promising tools for spatially resolved RNA modification analysis at the single-cell level.

## 3. Computational and Bioinformatics Approaches

Recent advances in epitranscriptomic bioinformatics have yielded a diverse ecosystem of computational tools (Table 4) for detecting, quantifying, and characterizing RNA modifications from high-throughput sequencing data, with particular emphasis on the unique signal properties of Oxford Nanopore Technologies (ONT) direct RNA sequencing (DRS). Statistical frameworks such as xPore model raw ionic current variation using multi-sample mixture models to identify differential modification events and estimate site-specific stoichiometry across conditions, but they typically require biological replicates and sufficient read depth for robust inference [140]. In contrast, Nanocompore performs largely model-free comparisons of signal distributions between modified and control samples, providing flexibility without predefined training data, while depending critically on high-quality hypomodified or unmodified reference datasets, adequate coverage, and careful experimental design [136].

Supervised, feature-based approaches—including m6Anet and EpiNano—utilize basecalling errors and machine-learning models to map transcriptome-wide m^6^A, yet their performance can be sensitive to the choice of training datasets, sequence context, and rapid updates in basecalling algorithms [141]. More general frameworks such as JACUSA2 integrate mismatch, indel, and reverse-transcription signatures across both Illumina and ONT platforms, enabling multi-modification detection while remaining susceptible to protocol-dependent and sequence-context biases [142].

Additional signal- and error-based tools, including ELIGOS, Tombo, Nanopolish, and nanoRMS, provide fine-grained access to raw current traces and single-molecule information for custom modification calling and stoichiometry estimation [143]. These methods, however, often demand substantial sequencing depth and involve considerable analytical complexity. Emerging deep-learning models such as TandemMod and ModiDeC attempt to integrate heterogeneous features or jointly model multiple modification types, but must continuously adapt to evolving nanopore chemistries, pore versions, and basecalling models that can challenge generalizability [144,145].

Complementing these analytical pipelines, resources such as RMPore aggregate site-level and molecule-level modification calls into curated reference datasets that support benchmarking and cross-method comparisons, while inevitably inheriting the biases and limitations of contributing studies [146]. Collectively, these tools illustrate a rapidly advancing computational landscape in which platform-specific capabilities, supported modification types, and underlying statistical or machine-learning strategies must be carefully matched to experimental design. Persistent challenges—including variable training data quality, sequencing depth requirements, and the intrinsic biological complexity of RNA modification landscapes—underscore the need for standardized benchmarking and continued methodological innovation.

## 4. Conclusions and Future Perspectives

Quantifying RNA modifications remains a major technical hurdle because of their chemical diversity, dynamic regulation, and frequent co-occurrence on the same transcript or even within the same local nucleotide environment. Mass spectrometry (MS)–based methods provide exceptional sensitivity and chemical specificity and therefore remain the de facto gold standard, but variability in implementation still limits quantitative comparability across laboratories. Addressing this gap will require community-accepted reference materials, synthetic multi-modified RNA standards, and robust benchmarking frameworks that explicitly assess performance for both single and combinatorial modification readouts. A central challenge is resolving combinatorial “modification barcodes,” including how marks such as m6A, Ψ, and A-to-I jointly influence RNA structure, translation, and decay in physiologically relevant settings. Integrating these epitranscriptomic layers with orthogonal transcriptomic, proteomic, and metabolic measurements is an essential next step for connecting modification patterns to phenotype in complex systems. Despite their strengths, MS workflows differ substantially in sample preparation, enzymatic hydrolysis, chromatographic separation, and data processing, which hampers direct comparison of quantitative measurements across laboratories. This variability highlights an urgent need for standardized reference materials, well-characterized synthetic RNAs carrying defined combinations of modifications, and shared benchmarking frameworks to evaluate and harmonize performance for both individual and combinatorial RNA modification measurements [147,148,149].

Immunoassays such as dot blot and ELISA offer accessible alternatives for detecting RNA modifications with minimal sample processing and lower equipment demands. However, their multiplexing capacity remains limited. Recent multiplexed immunoassay platforms, including Luminex^®^ and PINCER^®^, allow simultaneous quantification of multiple targets within a single reaction. The Luminex system employs fluorescently coded microspheres coated with target-specific antibodies, analyzed via dual-laser flow cytometry [150,151]. Although highly sensitive, this approach requires specialized instrumentation (e.g., Luminex 200™ or FLEXMAP 3D^®^) and expertise in data analysis. In contrast, the PINCER^®^ platform uses fluorescence resonance energy transfer (FRET) to detect target molecules with comparable sensitivity, without the need for complex sample preparation or specialized equipment. This assay involves a single mixing step followed by a ~30 min incubation, generating a robust and stable signal detectable with standard fluorescent plate readers [152]. Its simplicity and adaptability make it well-suited for high-throughput RNA modification studies. Nevertheless, the effectiveness of all immunoassays relies on the availability of high-affinity, modification-specific antibodies. Advances in antibody technology, including phage display and computational design, are expected to further improve specificity and affinity, expanding their utility in RNA modification research.

Rapid progress in sequencing-based methodologies has expanded the toolkit for mapping RNA modifications but continues to be constrained by sequence biases, enrichment artifacts, and incomplete stoichiometric information. Direct RNA sequencing retains native chemical information yet requires high depth and sophisticated error-correction algorithms. Antibody-, enzyme-, and chemistry-assisted approaches provide complementary sensitivity but face inherent biases and quantification limits. Critically, resolving co-occurring modifications on the same RNA molecule—an essential step for understanding modification crosstalk—remains difficult for most existing technologies. Integrating epitranscriptomic data with transcriptomic, proteomic, and metabolomic datasets also represents a major unmet need, as current multi-omics frameworks struggle to capture the combinatorial complexity and context specificity of RNA modification networks.

Computational and bioinformatic tools now play a central role in RNA modification discovery, particularly for base-calling, signal deconvolution in nanopore sequencing, peak calling in enrichment-based assays, and machine-learning–based prediction of putative modification sites. However, variability in algorithmic models, limited training datasets, and the absence of standardized benchmarking pipelines contribute to inconsistent performance across studies. Establishing unified evaluation frameworks and publicly accessible, high-quality reference datasets will be essential for method comparison, validation, and reproducibility.

Single-cell and spatially resolved epitranscriptomic technologies remain in early stages. Although single-cell sequencing and direct RNA detection platforms have begun to map modifications such as m^6^A and inosine at high resolution, challenges—including low coverage, high cost, data dropout, and limited compatibility with diverse modification types—currently restrict their broad application. Integration with spatial transcriptomics is further hindered by difficulties in preserving native RNA chemistry during tissue processing, as well as by limitations in imaging sensitivity, multiplexing capacity, and quantification accuracy. Addressing these technical barriers will be critical for understanding how modifications contribute to cellular heterogeneity and tissue organization.

Emerging nanopore direct RNA sequencing and structural, CRISPR-based, and imaging modalities illustrate the breadth of approaches now being applied to the epitranscriptome. Nanopore platforms uniquely allow multi-modification detection on single molecules and across full-length isoforms yet still face challenges in base-calling accuracy for rare or low-abundance modifications, context-dependent signal noise, and the scarcity of training sets that include defined combinations of marks. Future efforts should focus on improved pore chemistry and electronics, noise reduction and drift-correction strategies, and specialized base-calling models trained on well-characterized, multi-modified standards.

The technologies discussed in this review highlight the diverse strategies available for characterizing RNA and its modifications. In addition to established analytical and sequencing-based methods, several emerging approaches are rapidly advancing the field. Structural biology techniques such as cryo-electron microscopy (cryo-EM) [153,154,155] and X-ray crystallography [156,157] now enable atomic-resolution visualization of RNA structures and their chemical modifications. CRISPR-based systems are also expanding the toolkit for targeted manipulation of RNA, with catalytically inactive Cas13 (dCas13) being applied to A-to-I editing [158] and site-specific m^6^A installation [159]. Complementary imaging platforms, including CRISPR/dCas9–MS2-based RCasFISH, further improve the sensitivity of RNA detection in cells and tissue samples [160].

Across all detection modalities, accurate biological interpretation requires rigorous orthogonal validation. Many approaches yield putative modification sites that must be confirmed using independent methods such as mass spectrometry, genetic perturbation, or targeted biochemical assays. Establishing functional relevance, particularly in disease contexts—remains challenging due to the dynamic, context-dependent nature of modification turnover, distribution, and interactions with RNA-binding proteins.

Looking forward, several key gaps must be addressed to fully elucidate the biological significance of RNA modifications. These include the development of non-destructive, real-time detection technologies; improved nanopore base-calling models trained specifically on rare and low-abundance modifications; and tools capable of quantifying modification turnover rates in vivo with high temporal resolution. Additionally, the creation of standardized reference materials, comprehensive and interoperable modification databases, and harmonized computational pipelines will greatly enhance reproducibility and scalability. Integrating orthogonal analytical approaches with multi-omics datasets, structural biology techniques, CRISPR-based perturbation systems, and advanced imaging platforms will be essential for characterizing crosstalk modification and deciphering the multilayered regulatory logic of the epitranscriptome.

Collectively, the diverse technologies described in this review highlight both the progress made and the significant challenges that remain (Table 5). Continued innovation in analytical chemistry, sequencing technologies, structural biology, and computational biology will be necessary to achieve sensitive, specific, and high-throughput characterization of RNA modifications. These advances will accelerate the translation of epitranscriptomic insights into new diagnostic tools, therapeutic strategies, and biotechnological applications.

## Figures and Tables

**Table 1:** Antibody-Based Methods for RNA Modification Quantification.

Method	Subtype	Mechanism	Examples Studied	References
Dot Blot	–	RNA is spotted onto a membrane and incubated with a modification-specific antibody; detection is achieved via a secondary antibody.	Ψ, m^5^C, hm^5^C, m^6^A	[10,11,12,13,14,15,16]
ELISA	–	RNA samples compete with immobilized modified RNA for binding to modification-specific antibodies; bound antibodies are detected via colorimetric or fluorescent signals.	m^6^A, m^1^A, m^5^C, I, m^1^I	[17,18]
Antibody-Based Enrichment + NGS	m^6^A-seq/MeRIP-seq	RNA is fragmented and enriched using modification-specific antibodies, followed by sequencing.	m^6^A, m^1^A, hm^5^C, ac^4^C, m^7^G	[19,20]
	m^6^A-CLIP/miCLIP	Uses UV crosslinking to achieve single-nucleotide resolution.	m^6^A, m^1^A, hm^5^C, ac^4^C, m^7^G	[21,22,23]
	m^1^A-ID-seq/m^1^A-MAP/m^1^A-seq	Enrichment followed by sequencing to detect m^1^A modifications.	m^1^A	[24,25,26,27,28,29]
Antibody-Based Single-Cell Imaging	picoMeRIP-seq	Optimized MeRIP-seq for single-cell or rare cell types, improving sample recovery and signal-to-noise ratio.	m^6^A	[30]
	m^6^AISH-PLA	FISH probe hybridizes near the m^6^A site; secondary antibody complex + rolling circle amplification enables fluorescent detection.	m^6^A	[31]
	PREEM	Combines “AND” Boolean logic recognition with CAD-HCR amplification to detect multiple m^6^A sites simultaneously in single cells.	m^6^A	[32]

**Table 3:** Key Feature Comparison Between Antibody-Based and Non-Antibody Approaches.

Feature	Antibody-Based	Non-Antibody-Based
Input Requirement	ng–µg	ng–µg (varies)
Multiplexing	Limited (except advanced platforms)	High (MS, NGS, chemical)
Spatial Information	Possible (imaging, PLA)	Rare
Throughput	Moderate	High (MS, NGS, Nanopore)
Quantitative Accuracy	Moderate	High (MS, chemical-assisted NGS)
Limitations	Antibody specificity, enrichment bias	Harsh conditions, instrumentation, data analysis

**Table 4:** Computational tools for epitranscriptomic analysis.

Tool	Platform	Modification Type(s)	Unique Features	Limitations
xPore	ONT DRS	Various (m^6^A, others)	Statistical model for differential modification detection; stoichiometry estimation	Requires replicates and adequate coverage; limited to modifications that alter signal significantly
Nanocompore	ONT DRS	Various	Raw signal comparison between conditions; no supervised training required	Needs unmodified/control sample; sensitivity drops with low coverage; positional precision varies
m6Anet	ONT DRS	m^6^A	Multiple instance learning; transcriptome-wide m^6^A detection and stoichiometry	Focused only on m^6^A; dependent on training dataset and basecaller
EpiNano	ONT DRS	m^6^A	Uses basecalling errors and engineered features; supports custom training	Mainly tuned for m^6^A; performance affected by basecaller changes
JACUSA2	Illumina/ONT	Various	Detects mismatch, indels, RT signatures; integrates multiple library types	Signature-based; may be confounded by sequence or library prep biases
ELIGOS	ONT DRS	Various	Compares error profiles with controls to infer modifications	Sensitive to basecaller, coverage, and sequence context; high-depth requirement
Tombo	ONT DRS	Various	Resquiggle raw signal; flexible for custom analyses	Lower specificity without robust training or controls; resquiggling can fail in some regions
Nanopolish	ONT DRS	Various	Raw signal access for modification detection and downstream analysis	Dependent on chemistry and basecaller; limited modification specificity
nanoRMS	ONT DRS	Various	Read-level classification; per-site stoichiometry estimation	Requires high read depth; pipeline complexity; dependent on training data
TandemMod	ONT DRS	Multiple	Deep-learning framework for multi-mod detection via transfer learning	Sensitive to basecaller/chemistry changes; early-stage tool
ModiDeC	ONT DRS	Multiple	Modular classifier; extensible to new modifications	Real-world robustness not fully established
RMPore	ONT DRS (database)	Various	Aggregated single-molecule modification calls; benchmarking resource	Inherits biases of contributing datasets; absence of calls ≠ absence of modifications

**Table 5:** Comparison of RNA Modification Detection and Quantification Methods.

Category	Method	Principle	Advantages	Resolution	Typical Applications	Sensitivity	Specificity/Modification Coverage	Quantitative Accuracy	Limitations/Biases
>Antibody-based	Dot blot/Immuno-Northern blot	RNA immobilized on membrane; probed with modification-specific antibodies	Simple, inexpensive, fast	Global modification level	Detecting global levels of m^6^A, m^5^C, Ψ	Moderate (pmol-nmol)	Limited; depends on antibody cross-reactivity	Low–Moderate	Semi-quantitative; no site info; antibody-dependent. Antibody bias
	ELISA	Modified RNA competes with coated standards for antibody binding	Quantitative; easy; low RNA input	Global modification level.	Global quantification of m^6^A, m^1^A, m^5^C	Moderate (pmol-nmol)	Moderate; affected by antibody and matrix effects	Moderate	Cross-reactivity; limited dynamic range.
	Immunoprecipitation + NGS (MeRIP-seq, miCLIP)	Antibody enriches modified RNA fragments for sequencing	Transcriptome-wide mapping; single-nucleotide resolution possible	Transcrip-tome-wide mapping; sin-gle-nucleotide resolu-tion possible (miCLIP)	Mapping m^6^A, m^1^A, m^7^G, ac^4^C, hm^5^C	Moderate (pmol-nmol)	Often limited; many false positives from off-target binding	Moderate–High (relative)	Antibody bias; fragmentation bias; needs high input; Peak-calling bias (MeRIP-seq); UV damage and crosslinking efficiency bias (miCLIP)
	Antibody-based Imaging (picoMeRIP-seq, m6A-ISH-PLA, PREEM)	Fluorescent or proximity ligation imaging	Single-cell/molecule resolution; spatial visualization	Single-cell/molecule resolution	Visualizing m^6^A and other marks in single cells/tissues	Moderate (pmol-nmol)	Moderate; driven by probe and antibody design	Semi-quantitative	Antibody bias; Low throughput; technically demanding.
Non-antibody-based	Capillary Electrophoresis (CE/CE–MS)	Separation by charge/mass in capillary; detection via UV/LIF or MS	High separation efficiency; fast	Global modification level.	Detection of Ψ, I, m^2^G, m^1^A, m^6^A, m^5^C	Moderate (pmol-nmol)	High for well-resolved peaks; co-migration lowers it	Moderate	Reproducibility issues; moderate sensitivity. Digestion bias; Ionization bias in CE–MS
	Thin-layer Chromatography (TLC/2D-TLC/SCARLET)	Radiolabeled nucleotides separated by mobility on plates	Simple, low-cost	Global modification level (TLC/2D-TLC); Single-nucleotide resolution (SCARLET)	Site-specific quantification of m^6^A, Ψ	Moderate (pmol-nmol)	High when sequence/site is predefined (SCARLET)	Low–Moderate	Radioactive; low precision; prior sequence needed; digestion bias.
	PCR-based (SELECT, RTL-P, ligation-qPCR)	RNA modifications alter RT/ligase efficiency; quantified via qPCR	Sensitive, specific; low input	Single-nucleotide resolution	Site-specific quantification of m^6^A, m^1^A, Nm, Ψ	High	High when probe/primer design is optimal	High (relative)	Needs sequence knowledge; indirect detection; ligase discrimination bias; reverse transcriptase bias
	NMR Spectroscopy	Measures chemical shifts from modified nucleotides	Non-destructive; structural & dynamic insights	Single-nucleotide resolution possible.	Structural studies; effects of m^6^A, Ψ, D, m^1^A on RNA folding	Low	High	Moderate	Low sensitivity; requires large, pure RNA; structural bias.
	NGS-based (chemical/enzyme-assisted)	Chemical/enzymatic treatment induces RT signatures	High-throughput; single-nucleotide resolution	Transcrip-tome-wide mapping; Single-nucleotide resolution	Transcriptome-wide mapping (Pseudoseq, ac^4^C-seq, GLORI, DART-seq)	High	Often high but depends on reaction specificity and RT signature interpretation	Moderate–High	Often harsh chemicals; high RNA input; computationally complex.
	Nanopore Direct RNA Sequencing (DRS)	Direct sequencing of native RNA; detects current changes	Direct, long-read, real-time; no cDNA	Transcrip-tome-wide mapping; Single-nucleotide resolution	Global detection of m^6^A, m^5^C, Ψ, m^7^G	Moderate (pmol-nmol)	Moderate–high; model- and context-dependent	Moderate	High error rate; expensive; large input required.
	Non-antibody Imaging (ARPLA, DART-FISH)	Aptamer or deaminase labeling + FISH	Single-cell/molecule imaging; multiplex	Single-cell/molecule resolution	Visualizing m^6^A or glycoRNA localization	Moderate (pmol-nmol)	Limited; target-specific	Semi-quantitative	Lower throughput; partial transcript coverage. structure/accessibility bias
Mass Spectrometry (MS)-based	Bottom-up MS (LC–MS, LC–MS/MS)	RNA digested into nucleosides or short oligos before MS detection	Highly sensitive; detects multiple modifications simultaneously	Localize modification to a digested fragment.	Quantification of m^6^A, m^5^C, Ψ, etc., in mRNA, rRNA, tRNA, clinical samples	High (femtomole–picomole range)	High; mass/fragment–based discrimination	High, absolute quantification possible	Loses sequence context; isomer resolution challenging; digestion bias; ionization bias; fragmentation bias; modification-stability bias
	Top-down MS (FT-ICR, QqTOF)	Intact RNA molecules analyzed directly	Preserves sequence context; detects multiple modifications per molecule	Single-nucleotide resolution possible when fragmentation covers every backbone bond.	Structural analysis; site-specific mapping in small RNAs	Moderate–High (low pmol)	Multiple modifications in same molecule	Moderate–High	Requires pure RNA; complex spectra; limited throughput, ionization bias; fragmentation bias; modification-stability bias

## Data Availability

No new data were created or analyzed in this study. Data sharing is not applicable to this article.
